# Time Perception for Musical Rhythms: Sensorimotor Perspectives on Entrainment, Simulation, and Prediction

**DOI:** 10.3389/fnint.2022.916220

**Published:** 2022-07-05

**Authors:** Jessica M. Ross, Ramesh Balasubramaniam

**Affiliations:** ^1^Veterans Affairs Palo Alto Healthcare System and the Sierra Pacific Mental Illness, Research, Education, and Clinical Center, Palo Alto, CA, United States; ^2^Department of Psychiatry and Behavioral Sciences, Stanford University Medical Center, Stanford, CA, United States; ^3^Berenson-Allen Center for Non-invasive Brain Stimulation, Beth Israel Deaconess Medical Center, Boston, MA, United States; ^4^Department of Neurology, Harvard Medical School, Boston, MA, United States; ^5^Cognitive and Information Sciences, University of California, Merced, Merced, CA, United States

**Keywords:** sensorimotor timing, rhythm and beat perception, entrainment, simulation, shadowing, prediction

## Abstract

Neural mechanisms supporting time perception in continuously changing sensory environments may be relevant to a broader understanding of how the human brain utilizes time in cognition and action. In this review, we describe current theories of sensorimotor engagement in the support of subsecond timing. We focus on musical timing due to the extensive literature surrounding movement with and perception of musical rhythms. First, we define commonly used but ambiguous concepts including neural entrainment, simulation, and prediction in the context of musical timing. Next, we summarize the literature on sensorimotor timing during perception and performance and describe current theories of sensorimotor engagement in the support of subsecond timing. We review the evidence supporting that sensorimotor engagement is critical in accurate time perception. Finally, potential clinical implications for a sensorimotor perspective of timing are highlighted.

## Introduction

Music makes us move (Repp, 2005a,b; Janata et al., 2012; Iversen and Balasubramaniam, 2016; Ross et al., 2016a). But the more surprising finding is the phenomenon that movement planning networks are active when we listen to musical rhythms in the absence of any overt movement (Grahn and Brett, 2007, 2009; Chen et al., 2008a; Bengtsson et al., 2009; Iversen et al., 2009; Stupacher et al., 2013; Kasdan et al., 2022). Further, musical rhythms spontaneously modulate human brain excitability across sensory networks and movement planning networks (Repp, 2005a,b; Janata et al., 2012; Iversen and Balasubramaniam, 2016; Ross et al., 2016a). While there is a long history of study in how sensory systems inform action, there is less on how motor planning informs perception even though there is mounting evidence for bi-directionality between the systems. Control theory can be used to describe this bidirectionality of sensory and motor processes as a dynamical system, with internal forward models making predictions about sensory consequences of motor acts and those predictions guiding action and scaffolding perception (Prinz, 1997; Wolpert et al., 2009).

Sensorimotor frameworks that incorporate bidirectional sound-motor mappings contribute to comprehensive models of how the human brain uses and structures time (Schubotz, 2007; Merchant and Honing, 2014; Patel and Iversen, 2014; Morillon and Baillet, 2017) and are critical for understanding human perception of time at the sub-second scale (Ross et al., 2016b; Cook et al., 2022). In this mini-review, we focus on the role that the human motor system plays in the perception of time by drawing from recent evidence in behavioral and neural studies of rhythm.

**FIGURE 1:**
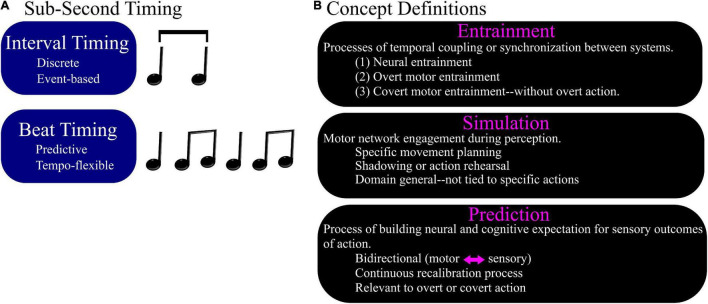
Timing for musical rhythm perception. **(A)** Sub-second timing can be discrete, such as for perception of interval durations, or can be continuous, as in the case of musical beat perception. Musical timing co-opts sensorimotor systems for accurate continuous timing perception. Evidence from overt motor synchronization tasks supports that musical timing is predictive and tempo-flexible. **(B)** Definition of concepts in the context of sensorimotor neuroscience: entrainment, simulation, prediction.

One important caveat is that perception of longer durations (>1 s) may rely more on memory and be more consistent with internal clock models (Staddon, 2005), but perception of sub-second intervals may be influenced more by distributed “state dependency” (Buonomano and Merzenich, 1995) and therefore more susceptible to mediation by sensory expectation and attention (Large and Jones, 1999; Eagleman, 2005; Hurley et al., 2018). However, all sub-second intervals do not require the same level of sensorimotor engagement. For example, sub-second intervals that are embedded in complex musical rhythms rely on predictive mechanisms that are distinct from the mechanisms of absolute interval timing (Teki et al., 2011, 2012; Patel and Iversen, 2014; Iversen and Balasubramaniam, 2016; Ross et al., 2016b). Absolute interval timing between auditory events may rely on “interval” timing mechanisms and music may require “beat” timing, a continuous process that involves finding the underlying pulse in auditory events with some rhythmicity (Figure 1A).

In this review, we discuss the role of motor regions of the brain in accurate time perception, specifically in the context of music. First, we define the concepts of entrainment, simulation, and prediction when used in the context of time and event perception. We then summarize the literature on sensorimotor timing that uses overt motor tasks and go on to describe current theories of sensorimotor engagement in the support of subsecond timing even in the absence of overt motor actions. Lastly, because this phenomenon of sensorimotor support of sub-second timing can be esoteric in concept, we discuss real world implications (Thaut et al., 1996; Altenmüller and Schlaug, 2013; Nombela et al., 2013; Ventura et al., 2016) for investigation of this brain process which is relevant across the lifespan (Kuhl et al., 2014), across cultures (Madison, 2006; Madison et al., 2011; Janata et al., 2012), with important implications for evolutionary processes (Patel et al., 2009; Merchant and Honing, 2014; Patel, 2018).

## Definition of Concepts From Sensorimotor Neuroscience

Key concepts relevant to the topic of sensorimotor timing include entrainment, simulation, and prediction. Although commonly used both colloquially and in academic writing, these concepts can be ambiguous and/or inconsistently defined (Cohen and Gulbinaite, 2014). Here, before describing current theoretical models of sensorimotor engagement, we provide concise definitions relevant to this context (Figure 1B).

### Entrainment

Describes processes of temporal coupling or synchronization between two independent oscillatory systems by virtue of phase alignment. Three primary uses of entrainment in the context of sensorimotor timing include (1) neural entrainment, (2) overt motor entrainment, and (3) covert motor entrainment (without overt action). Neural entrainment characterizes coupling between sensory stimuli and any neural oscillation as measured by electroencephalography (EEG) or magnetoencephalography (MEG) (Nozaradan et al., 2011, 2012). Overt motor entrainment is coupling between body movements and sensory stimuli, such as musical rhythms (Balasubramaniam, 2005; Repp, 2005b; Keller and Repp, 2008; Repp and Su, 2013; Pabst and Balasubramaniam, 2018).

Measuring motor entrainment is useful for understanding movement dynamics such as variability, stability, and adaptability of entrainment, coordination between multiple effectors, and the neural basis of rhythmic timekeeping (Ross and Balasubramaniam, 2014). Finally, covert motor entrainment is a type of neural entrainment but refers specifically to coupling between sensory stimuli and neural oscillations supporting body movement, but without execution of movement (Repp, 2005b). Bruno Repp suggested that perception of auditory rhythms relies on covert action—that synchronizing with a sequence is not so different than simply perceiving a sequence without moving along with it (Repp, 2005a,b). It is unknown to what degree covert motor entrainment reflects “shadowing” or “mirroring” of sensory sampling in the auditory system (Ross et al., 2016b), but accumulating evidence supports that motor networks also have a more causal or predictive role in auditory time perception without which human musical beat perception would be impaired (Grahn and Brett, 2009; Iversen et al., 2009; Grube et al., 2010b,a; Ross et al., 2018).

### Simulation

Describes motor network engagement during perception. Simulation can be specific movement planning (Miyake, 1902; Woodrow, 1932; Aschersleben et al., 2001; Drewing et al., 2002; Repp and Su, 2013), shadowing or action rehearsal (Miall, 2003; Tian and Poeppel, 2010; Pfordresher, 2011; Press and Cook, 2015), or can be more domain general and not tied to specific actions (Prinz, 1997; Schubotz, 2007; Shin et al., 2010). Many theories attempt to explain the role of simulation in perception (Balasubramaniam et al., 2021), but the scope of concepts elicited under an umbrella of simulation is quite broad. The reason for this could be due to limited conclusive evidence supporting any one proposed role for motor network engagement during perception. However, simulation supporting musical beat perception has developed more focus due to accumulating neurophysiological supporting evidence for the proposed roles in auditory timing perception (Schubotz, 2007; Merchant and Honing, 2014; Patel and Iversen, 2014; Morillon and Baillet, 2017).

### Prediction

The process of building neural and cognitive expectations for sensory outcomes of action. Prediction is a critical concept in models of sensorimotor interaction and is connected necessarily to error correction when there are discrepancies between the predicted and actual sensory feedback from action (Lombard, 1911; Prinz, 1997; Wolpert and Kawato, 1998; Miall, 2003; Pfordresher and Mantell, 2009; Wolpert et al., 2009; Tian and Poeppel, 2010; Pfordresher, 2011; Zollinger and Brumm, 2011; Therrien et al., 2012; Wolpert and Flanagan, 2016; Yang et al., 2016). Models that describe prediction and error correction as a continuously updated process of recalibration of internal models best account for experimental data (Phillips-Silver and Trainor, 2005, 2007; Grahn and Brett, 2007; Iversen et al., 2009; Manning and Schutz, 2013; Blecher et al., 2016; Kotz et al., 2016). Prediction is also used to describe bidirectional and continuous recalibration of sensory experience during covert action, such as in the case of covert motor simulation for musical beat perception (Schubotz, 2007; Patel and Iversen, 2014). Although the biological processes underlying sensory prediction are still being investigated, experimental data supports that such predictions do occur in the support of both overt and covert movement planning.

While entrainment describes phase coupling between systems, for example pendulums that go into synchrony when coupled can be viewed as a classic example of physical entrainment (Stepp and Turvey, 2017), prediction, is an active process that reflects the generation of cognitive, sensory, or motor expectations in neural/biological systems (Ross and Balasubramaniam, 2014). It may be the case that entrainment could support the generation or maintenance of predictions, but as we argue, the concepts are distinct. There is growing evidence for how cerebral networks may support the generation and recalibration of sensory predictions, and modeling work that can be used to generate testable hypotheses with regard to the underlying mechanisms of prediction. Neural signatures of predictive oscillatory phase alignment will be reviewed in detail below, both during overt and covert entrainment.

In the following section, we detail how entrainment, simulation, and prediction get instantiated in neural systems when exposed to rhythmic auditory sensory information.

## Top-Down and Bottom-Up Contributions to Overt Motor Entrainment

Much of the literature on sub-second timing comes from motor entrainment tasks (Balasubramaniam, 2005; Repp, 2005b; Keller and Repp, 2008; Repp and Su, 2013; Pabst and Balasubramaniam, 2018), often with finger-tapping synchronization to an auditory stimulus. For example, empirical studies of overt motor entrainment to auditory rhythms suggest that beat perception involves perceptual constructs of timing (Miyake, 1902; Woodrow, 1932; Repp, 2005b). When asked to tap a finger in time with an auditory rhythm, precise timing analyses show that people often tap just prior to the beat time. This phenomenon of “entrained” taps preceding the auditory events is an effect called *negative mean asynchrony* (Miyake, 1902). Some theories to explain this phenomenon suggest that the asynchronies occur because actions are planned using the perceivable results of these actions (Prinz, 1990, 1997). Because tactile/kinesthetic sensation has longer nerve conduction and central processing times than auditory sensation, the actual tap events must occur prior to the actual auditory events for the perceptual effects of the events to be aligned in time (Aschersleben and Prinz, 1995). Other theories focus more on timing error minimization by undershooting interval durations (Vorberg and Wing, 1996) or by asymmetric evaluation of early versus late errors (Vos and Helsper, 1992). However, all proposed explanations require spontaneously generated perceptual constructs (Aschersleben et al., 2001; Drewing et al., 2002) and other top-down strategies (Repp and Su, 2013; Pabst and Balasubramaniam, 2018).

Although these overt motor entrainment tasks have provided foundational insights into human mechanisms of timing, the tasks themselves may influence timing. For instance, different timing strategies may be elicited by the type of sensory feedback in a task—discrete events may elicit ‘event’ timing mechanisms and continuous sensory feedback during the task may elicit more continuous timing mechanisms (Iversen and Balasubramaniam, 2016). Timing can be influenced by motor involvement—sensorimotor entrainment is influenced by “state” of the motor effector (i.e., effector position, sensory feedback, and state estimation) (Balasubramaniam et al., 2004; Torre and Balasubramaniam, 2009; Ross and Balasubramaniam, 2014; Pabst and Balasubramaniam, 2018). Another aspect of beat-based timing that we learn from overt motor entrainment is that rhythm perception is tempo flexible–Precise predictions are flexible to rhythms that speed up and slow down. Changes to tempo, at least within a range of 94–176 beats per minute, do not have a negative impact on perception of rhythmic structure or the underlying musical beat (Hanson et al., 1971; van Noorden and Moelants, 1999; London, 2004; McAuley et al., 2006; Su and Pöppel, 2012; Patel and Iversen, 2014). These results all suggest that timing mechanisms in overt motor entrainment tasks not only reflect top-down timing constructs but also bottom-up incorporation. Overt motor entrainment relies on a continuous and bidirectional relationship between perceptual constructs of time and error (Repp, 2005b; Repp and Su, 2013; Iversen and Balasubramaniam, 2016). Psychophysical and neural studies of time perception support this notion, that timing is mediated by top-down processes while accounting for bottom-up information (Large and Jones, 1999; Eagleman, 2005; Hurley et al., 2018).

Because we must plan for a synchronized movement in advance, and there is some automaticity to this planning when we listen to auditory rhythms, it is reasonable to ask whether we also perform some degree of motor planning every time we perceive a rhythm even if we do not move any body part in time with it. Musical rhythms can be used to learn about neural signatures of and substrates for timing (Teki et al., 2011, 2012; Arnal, 2012; Morillon and Baillet, 2017). Musical rhythms are complex, hierarchical patterns of auditory events that induce perceptual constructs of timing and engage motor networks in the brain. Distributed network involvement for time perception is not a byproduct of a motor task but instead timing mechanisms can co-opt sensorimotor systems for accurate perception of time (Patel and Iversen, 2014; De Kock et al., 2021). In essence, musical timing co-opts mechanisms of sensorimotor timing (Balasubramaniam et al., 2021) and the result is more continuous than “event” timing (Figure 1A). In the section below, we discuss the most compelling evidence for this argument. First, that musical timing relies on perceptual constructs of time instead of only acoustic features. Second, that signatures of covert movement can be observed and manipulated using passive music listening experiments that do not involve overt movement.

## Sensorimotor Engagement When There is No Motor Task

To support the proposal that musical timing depends on continuous timing processes from co-opting of sensorimotor systems, evidence must show motor system engagement during musical timing perception with no motor action. It is critical for testing this account of musical timing that passive tasks are used that do not involve overt motor action (Grahn and Brett, 2007, 2009; Chen et al., 2008a; Bengtsson et al., 2009; Iversen et al., 2009; Stupacher et al., 2013; Kasdan et al., 2022). Imaging modalities such as functional magnetic resonance imaging (fMRI), MEG, and EEG can be used in place of finger-tapping to understand predictive timing without motor actions during passive music listening. fMRI during rhythm perception experiments consistently shows activation in areas of the brain that are known to be involved in movement of the body, and these areas include primary motor cortex, premotor cortices, the basal ganglia, supplementary motor area, and cerebellum (Grahn and Brett, 2007, 2009; Zatorre et al., 2007; Chen et al., 2008a,b). Covert motor activity during passive music listening presents consistently across studies, even with considerable stimulus variability. Interestingly, the stimulus variability shows up less in whether we see covert action and more in which motor networks are covertly activated (Gordon et al., 2018).

However, it is unknown why passive music listening engages sensorimotor networks. Several relevant proposals exist but rigorous testing is required to support or refute these proposals. For instance, domain-general theories, such as common-coding and ideomotor theory (Prinz, 1997; Shin et al., 2010), suggest that this covert movement is not critical to perception (Press and Cook, 2015), while other theories, such as active inference (Friston et al., 2011; Clark, 2015), predictive coding of rhythmic incongruity model (PCRI) (Vuust et al., 2018), computation through dynamics (Balasubramaniam et al., 2021), and dynamic attending theory (DAT) (Jones, 1976; Arnal, 2012) support that covert motor engagement may be causally involved with timing predictions (Bolton, 1894; Sperry, 1952; Prinz, 1997; Schubotz et al., 2000; Jeannerod, 2001; Repp, 2005b; Zatorre et al., 2007; Vuust et al., 2009; Rauschecker, 2011; Arnal, 2012; Ross et al., 2016b). One proposal that is of particular importance because it poses hypotheses that are empirically testable is the Action Simulation for Auditory Prediction Hypothesis (ASAP) (Patel and Iversen, 2014), which hypothesizes that interactions between motor planning and auditory perception are continuous and bidirectional [see “reentry;” (Edelman, 1989)], necessary for rhythm perception, and rely on dorsal auditory pathway projections in parietal cortex (Patel and Iversen, 2014; Patel, 2021). There is accumulating experimental evidence supporting the hypothesis that covert motor activation while listening to rhythms has a causal role in shaping rhythm perception (Repp, 2005b; Iversen et al., 2009; Niell and Stryker, 2010; Wekselblatt and Niell, 2015; Nozaradan et al., 2016; Ross et al., 2018), including cases of disease-related (Grahn and Brett, 2009; Grube et al., 2010a; Grahn and Rowe, 2013; Kotz et al., 2016) or stimulation-evoked (Pollok et al., 2008; Grube et al., 2010b; Ross et al., 2018) brain lesions in motor networks impairing perception. Network disruptions, such as those induced using non-invasive brain stimulation methods including Transcranial Magnetic Stimulation (TMS), can be used to safely test for causality and therefore support or refute specific causal hypotheses. When TMS is applied to the cerebellum, accurate interval timing, but not beat timing, is impaired (Grube et al., 2010b). When applied to dorsal stream areas proposed in ASAP such as parietal and premotor cortex, aspects of beat timing, but not interval timing, are impaired (Ross et al., 2018). These TMS studies provide causal evidence to support the specific hypothesis of ASAP that auditory-motor connectivity is necessary for rhythm perception.

One emerging paradigm is to have subjects listen passively to musical rhythms and measure the effects on neural entrainment of oscillatory activity recorded using MEG (Fujioka et al., 2009, 2015; Iversen et al., 2009). Brain oscillation recorded in MEG and EEG is a byproduct of fluctuations in synchronized neuronal population activity in the cortex. Measuring oscillatory brain dynamics can be revealing for understanding time-sensitive excitatory and inhibitory processes (Arnal and Giraud, 2012) and is often described within frequency bands of oscillation such as alpha (8–13 Hz), beta (13–30 Hz), and gamma (>30 Hz). Fujioka et al. (2009) showed during passive music listening induced beta and gamma phase dynamics from auditory cortices that desynchronized just prior to beat onset and synchronized after beat onset. A later MEG study showed the rate of beta desynchronization in anticipation of the beat is dependent on the tempo of the stimulus, whereas beta synchronization following the beat is consistent across multiple tempi (Fujioka et al., 2012). Authors additionally found cortico-cortical coherence that followed the tempo of the rhythms between auditory cortices and sensorimotor cortex, supplementary motor area (SMA), inferior frontal gyrus, and cerebellum (Fujioka et al., 2012). These phase dynamics are replicable (Iversen et al., 2009; Fujioka et al., 2012, 2015), strongest for musical stimuli with complex metrical hierarchy, follow metrical structures (Fujioka et al., 2015), and occur even when beats are not heard but imagined based on metrical expectations (Iversen et al., 2009). Musical rhythms with multiple metrical interpretations entrain neural oscillations differently depending on the meter perceived by the listener, and when the perception of meter changes, so does neural entrainment. Further, early auditory responses to beat are equivalent whether the result of imagined beats or non-imagined physical accents (Iversen et al., 2009). This work supports that perception of rhythms, with no motor task, entrains motor-related oscillatory phase dynamics.

**FIGURE 2:**
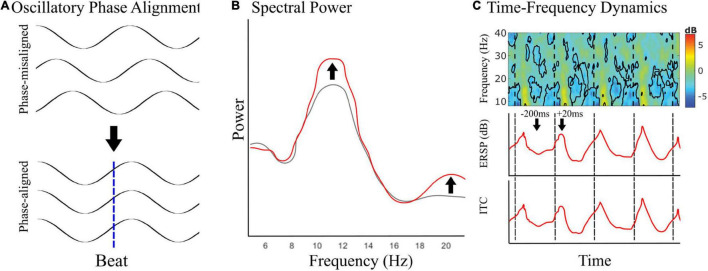
Covert motor engagement in EEG. **(A)** Schematic showing phase-alignment in motor-related oscillatory brain activity to the predicted musical beat times during passive music listening. **(B,C)** Signatures of covert motor engagement in electrophysiological recordings–spectral power changes (**B**, schematic) and time-frequency dynamics [**C**, in this example to rhythmic auditory events during passive listening as described by Comstock et al. (2021)]. Event-related spectral perturbation (ERSP) is used to observe averaged dynamic changes in amplitude of the broad band frequency spectrum as a function of time and captures phase shifts in ongoing oscillatory activity. Inter-trial coherence (ITC) describes how consistent oscillatory phase is across trials and can be used to quantify phase locking to an event. If the time course of averaged ERSP and ITC is the same, then the event is phase locking oscillations consistently across trials.

Oscillatory phase dynamics, previously shown only using MEG, have recently been investigated using EEG (Figure 2). Musical rhythms appear to entrain alpha oscillations that occur over sensorimotor cortices, commonly called mu (μ). In this work, EEG μ had sources localizing to premotor and motor cortices (Ross et al., 2022). This work suggests that covert movement during passive music listening may reflect fluctuations in motor cortical inhibition. In a recent study, Comstock et al. (2021) showed that there is network specificity to sensory rhythm-induced EEG beta entrainment that localizes to sensorimotor, occipital, parietal, and frontal networks. This work provides evidence for overlapping networks of predictive beta activity based on common activation in the parietal and right frontal regions, auditory-specific predictive beta in bilateral sensorimotor regions, and visually specific predictive beta in midline central, and bilateral temporal/parietal regions. Additionally, the authors find predictive beta activity in the left sensorimotor region specific to auditory rhythms. Overall, this work implicates modality-dependent networks for auditory and visual rhythm perception.

Inclusively, this work shows that beta and alpha neural oscillations can be phase aligned using musical rhythms (Fujioka et al., 2009, 2012, 2015; Comstock et al., 2021; Ross et al., 2022), which is consistent with the literature on sensory phase entrainment more broadly (Snyder and Large, 2005; Cardin et al., 2009; Arnal and Giraud, 2012; Santarnecchi et al., 2016; Iaccarino et al., 2018; Comstock et al., 2021; National Library of Medicine., 2021a,b; Ross et al., 2022). This growing literature shows that musical rhythms reliably induce phase synchronization (Snyder and Large, 2005; Fujioka et al., 2009, 2012, 2015; Iversen et al., 2009; Saleh et al., 2010; Varlet et al., 2020; Comstock et al., 2021; Ross et al., 2022) that is tempo dependent (Fujioka et al., 2012), can be caused by either heard or imagined stimuli (Snyder and Large, 2005; Iversen et al., 2009), and modulates network coherence (Fujioka et al., 2012). This work with musical rhythms supports ongoing mechanistic investigations into the roles of sensorimotor expectation for the timing of musical beats—The emerging narrative is that auditory timing prediction relies on strong interactions between motor systems and auditory cortices (Janata et al., 2012; Repp and Su, 2013; Iversen and Balasubramaniam, 2016; Ross et al., 2016a,b), possibly mediated through projections in parietal cortex (Patel and Iversen, 2014; Ross et al., 2018), and have signatures in frequency band-specific oscillatory activity (Comstock et al., 2021; Ross et al., 2022). Signatures of predictive phase alignment in EEG should be present in spectral power (Figure 2B) and in both event-related spectral perturbations (ERSP) and inter-trial coherence (ITC) of time-frequency dynamics (Figure 2C). Thus, covert motor activity can be induced, measured, localized, and shown to be predictive using a combination of passive music listening and electrophysiological recording, such as with MEG and EEG.

## Conclusion and Future Directions

We review the literature here that supports that motor networks, which are specialized for sensorimotor prediction and error correction for overt action, may also be causally involved in covert motor entrainment in the case of musical beat perception (Grahn and Brett, 2007, 2009; Grube et al., 2010b,a; Ross and Balasubramaniam, 2014; Iversen and Balasubramaniam, 2016; Ross et al., 2016b). Many theoretical models explaining the relationship from sensory events to action exist (Balasubramaniam et al., 2021) but the possibility that motor networks have a causal impact on sensory perception, and that the action-perception relationship is bidirectional, is not only theoretically compelling, but it contributes to an understanding that time perception can be an actively predictive and simulatory neural process (Prinz, 1997; Wolpert et al., 2009; Patel and Iversen, 2014; Balasubramaniam et al., 2021).

This perspective of time perception has numerous implications for topics of development, brain health, and motor rehabilitation. Atypical oscillatory activity is associated with cognitive deficits (Cardin et al., 2009; Santarnecchi et al., 2016) and disease (Koenig et al., 2005; Iaccarino et al., 2018; Benwell et al., 2020), including in fluid intelligence (Santarnecchi et al., 2016) and attention (Cardin et al., 2009), type 2 diabetes (Benwell et al., 2020), mild cognitive impairment (Koenig et al., 2005), and Alzheimer’s disease (Koenig et al., 2005; Iaccarino et al., 2018; Benwell et al., 2020). Modulation of these atypical oscillations is being explored for therapeutic effects using optogenetics (Cardin et al., 2009), tACS (Santarnecchi et al., 2016), and sensory stimuli in the gamma band (Cardin et al., 2009; National Library of Medicine., 2021a,b). Other applications for sensory-induced phase entrainment of neural oscillatory activity should be explored, including in beta and alpha bands. Beta and alpha bands are relevant to engagement of motor networks, and therefore are relevant for sensory and motor disorders (Saltuklaroglu et al., 2018). However, applications for musical sensorimotor timing critically rely on a more complete understanding of covert motor timing and what the neural substrate is supporting these processes (Patel and Iversen, 2014; Kasdan et al., 2022). We suggest that covert motor processes should be studied using methods that do not require overt action, and we provide some examples of signatures of motor-related oscillatory phase alignment in EEG. We are currently investigating how perturbing motor systems will influence the neural signatures of auditory predictive processes using combined TMS-EEG methods.

## Author Contributions

JR prepared figures. Both authors conceived, drafted, revised, and approved the submitted manuscript.

## Conflict of Interest

The authors declare that the research was conducted in the absence of any commercial or financial relationships that could be construed as a potential conflict of interest.

## Publisher’s Note

All claims expressed in this article are solely those of the authors and do not necessarily represent those of their affiliated organizations, or those of the publisher, the editors and the reviewers. Any product that may be evaluated in this article, or claim that may be made by its manufacturer, is not guaranteed or endorsed by the publisher.

## References

[B1] Altenmüller E. Schlaug G. (2013). Neurologic music therapy: the beneficial effects of music making on neurorehabilitation. *Acoust. Sci. Tech.* 34 5–12. 10.1250/ast.34.5

[B2] Arnal L. H. (2012). Predicting “When” Using the Motor System’s Beta-Band Oscillations. *Front. Hum. Neurosci.* 6:225. 10.3389/fnhum.2012.00225 22876228 PMC3410664

[B3] Arnal L. H. Giraud A.-L. (2012). Cortical oscillations and sensory predictions. *Trends Cogn. Sci.* 16 390–398. 10.1016/j.tics.2012.05.003 22682813

[B4] Aschersleben G. Gehrke J. Prinz W. (2001). Tapping with peripheral nerve block. *Exp. Brain Res.* 136 331–339. 10.1007/s002210000562 11243475

[B5] Aschersleben G. Prinz W. (1995). Synchronizing actions with events: the role of sensory information. *Percept. Psychophys.* 57 305–317. 10.3758/BF03213056 7770322

[B6] Balasubramaniam R. (2005). *Trajectory Formation in Timed Repetitive Movements. Progress in Motor Control IV.* New York, NY: Springer, 47–54.

[B7] Balasubramaniam R. Wing A. M. Daffertshofer A. (2004). Keeping with the beat: movement trajectories contribute to movement timing. *Exp. Brain Res.* 159, 129–134. 10.1007/s00221-004-2066-z 15365663

[B8] Balasubramaniam R. Haegens S. Jazayeri M. Merchant H. Sternad D. Song J.-H. (2021). Neural Encoding and Representation of Time for Sensorimotor Control and Learning. *J. Neurosci.* 41 866–872. 10.1523/JNEUROSCI.1652-20.2020 33380468 PMC7880297

[B9] Bengtsson S. L. Ullén F. Henrik Ehrsson H. Hashimoto T. Kito T. Naito E. (2009). Listening to rhythms activates motor and premotor cortices. *Cortex* 45 62–71. 10.1016/j.cortex.2008.07.002 19041965

[B10] Benwell C. S. Y. Davila-Pérez P. Fried P. J. Jones R. N. Travison T. G. Santarnecchi E. (2020). EEG spectral power abnormalities and their relationship with cognitive dysfunction in patients with Alzheimer’s disease and type 2 diabetes. *Neurobiol. Aging* 85 83–95. 10.1016/j.neurobiolaging.2019.10.004 31727363 PMC6942171

[B11] Blecher T. Tal I. Ben-Shachar M. (2016). White matter microstructural properties correlate with sensorimotor synchronization abilities. *NeuroImage* 138 1–12. 10.1016/j.neuroimage.2016.05.022 27165760

[B12] Bolton T. L. (1894). Rhythm. *Am. J. Psychol.* 6 145. 10.2307/1410948

[B13] Buonomano D. V. Merzenich M. M. (1995). Temporal Information Transformed into a Spatial Code by a Neural Network with Realistic Properties. *Science* 267 1028–1030. 10.1126/science.7863330 7863330

[B14] Cardin J. A. Carlén M. Meletis K. Knoblich U. Zhang F. Deisseroth K. (2009). Driving fast-spiking cells induces gamma rhythm and controls sensory responses. *Nature* 459 663–667. 10.1038/nature08002 19396156 PMC3655711

[B15] Chen J. L. Penhune V. B. Zatorre R. J. (2008a). Listening to Musical Rhythms Recruits Motor Regions of the Brain. *Cereb. Cortex* 18 2844–2854. 10.1093/cercor/bhn042 18388350

[B16] Chen J. L. Penhune V. B. Zatorre R. J. (2008b). Moving on Time: brain Network for Auditory-Motor Synchronization is Modulated by Rhythm Complexity and Musical Training. *J. Cogn. Neurosci.* 20 226–239. 10.1162/jocn.2008.20018 18275331

[B17] Clark A. (2015). “Embodied Prediction,” in *Open MIND*, eds Metzinger T. Windt J. M. (7(T). Frankfurt am Main: MIND Group), 10.15502/97839585701

[B18] Cohen M. X. Gulbinaite R. (2014). Five methodological challenges in cognitive electrophysiology. *NeuroImage* 85 702–710. 10.1016/j.neuroimage.2013.08.010 23954489

[B19] Comstock D. C. Ross J. M. Balasubramaniam R. (2021). Modality-specific frequency band activity during neural entrainment to auditory and visual rhythms. *Eur. J. Neurosci.* 54 4649–4669. 10.1111/ejn.15314 34008232

[B20] Cook J. R. Li H. Nguyen B. Huang H.-H. Mahdavian P. Kirchgessner M. A. (2022). Secondary auditory cortex mediates a sensorimotor mechanism for action timing. *Nat. Neurosci.* 25 330–344. 10.1038/s41593-022-01025-5 35260862 PMC9288832

[B21] De Kock R. Gladhill K. A. Ali M. N. Joiner W. M. Wiener M. (2021). How movements shape the perception of time. *Trends Cogn. Sci.* 25 950–963. 10.1016/j.tics.2021.08.002 34531138 PMC9991018

[B22] Drewing K. Hennings M. Aschersleben G. (2002). The contribution of tactile reafference to temporal regularity during bimanual finger tapping. *Psychol. Res.* 66 60–70. 10.1007/s004260100074 11963279

[B23] Eagleman D. M. (2005). Time and the Brain: how Subjective Time Relates to Neural Time. *J. Neurosci.* 25 10369–10371. 10.1523/JNEUROSCI.3487-05.2005 16280574 PMC6725822

[B24] Edelman C. M. (1989). *The Remembered Present: A Biological Theory of Consciousness.* New York, NY: Basic Books.

[B25] Friston K. Mattout J. Kilner J. (2011). Action understanding and active inference. *Biol. Cybern.* 104 137–160. 10.1007/s00422-011-0424-z 21327826 PMC3491875

[B26] Fujioka T. Ross B. Trainor L. J. (2015). Beta-Band Oscillations Represent Auditory Beat and Its Metrical Hierarchy in Perception and Imagery. *J. Neurosci.* 35 15187–15198. 10.1523/JNEUROSCI.2397-15.2015 26558788 PMC6605356

[B27] Fujioka T. Trainor L. J. Large E. W. Ross B. (2009). Beta and Gamma Rhythms in Human Auditory Cortex during Musical Beat Processing. *Ann. N. Y. Acad. Sci.* 1169 89–92. 10.1111/j.1749-6632.2009.04779.x 19673759

[B28] Fujioka T. Trainor L. J. Large E. W. Ross B. (2012). Internalized Timing of Isochronous Sounds Is Represented in Neuromagnetic Beta Oscillations. *J. Neurosci.* 32 1791–1802. 10.1523/JNEUROSCI.4107-11.2012 22302818 PMC6703342

[B29] Gordon C. L. Cobb P. R. Balasubramaniam R. (2018). Recruitment of the motor system during music listening: an ALE meta-analysis of fMRI data. *PLoS One* 13:e0207213. 10.1371/journal.pone.0207213 30452442 PMC6242316

[B30] Grahn J. A. Brett M. (2007). Rhythm and Beat Perception in Motor Areas of the Brain. *J. Cogn. Neurosci.* 19 893–906. 10.1162/jocn.2007.19.5.893 17488212

[B31] Grahn J. A. Brett M. (2009). Impairment of beat-based rhythm discrimination in Parkinson’s disease. *Cortex* 45 54–61. 10.1016/j.cortex.2008.01.005 19027895

[B32] Grahn J. A. Rowe J. B. (2013). Finding and Feeling the Musical Beat: striatal Dissociations between Detection and Prediction of Regularity. *Cereb. Cortex* 23 913–921. 10.1093/cercor/bhs083 22499797 PMC3593578

[B33] Grube M. Cooper F. E. Chinnery P. F. Griffiths T. D. (2010a). Dissociation of duration-based and beat-based auditory timing in cerebellar degeneration. *Proc. Nat. Acad. Sci.* 107 11597–11601. 10.1073/pnas.0910473107 20534501 PMC2895141

[B34] Grube M. Lee K.-H. Griffiths T. D. Barker A. T. Woodruff P. W. (2010b). Transcranial magnetic theta-burst stimulation of the human cerebellum distinguishes absolute, duration-based from relative, beat-based perception of subsecond time intervals. *Front. Psychol.* 1:171. 10.3389/fpsyg.2010.00171 21833234 PMC3153783

[B35] Hanson F. E. Case J. F. Buck E. Buck J. (1971). Synchrony and Flash Entrainment in a New Guinea Firefly. *Science* 174 161–164. 10.1126/science.174.4005.161 17742039

[B36] Hurley B. K. Fink L. K. Janata P. (2018). Mapping the dynamic allocation of temporal attention in musical patterns. *J. Exp. Psychol. Hum. Percept. Perform.* 44 1694–1711. 10.1037/xhp0000563 30091636

[B37] Iaccarino H. F. Singer A. C. Martorell A. J. Rudenko A. Gao F. Gillingham T. Z. (2018). Author Correction: gamma frequency entrainment attenuates amyloid load and modifies microglia. *Nature* 562 E1–E1. 10.1038/s41586-018-0351-4 30046102

[B38] Iversen J. R. Balasubramaniam R. (2016). Synchronization and temporal processing. *Curr. Opin. Behav. Sci.* 8 175–180. 10.1016/j.cobeha.2016.02.027

[B39] Iversen J. R. Repp B. H. Patel A. D. (2009). Top-Down Control of Rhythm Perception Modulates Early Auditory Responses. *Ann. N. Y. Acad. Sci.* 1169 58–73. 10.1111/j.1749-6632.2009.04579.x 19673755

[B40] Janata P. Tomic S. T. Haberman J. M. (2012). Sensorimotor coupling in music and the psychology of the groove. *J. Exp. Psychol.Gen.* 141 54–75. 10.1037/a0024208 21767048

[B41] Jeannerod M. (2001). Neural Simulation of Action: a Unifying Mechanism for Motor Cognition. *NeuroImage* 14 S103–S109. 10.1006/nimg.2001.0832 11373140

[B42] Jones M. R. (1976). Time, our lost dimension: toward a new theory of perception, attention, and memory. *Psychol. Rev.* 83 323–355. 10.1037/0033-295X.83.5.323 794904

[B43] Kasdan A. V. Burgess A. N. Pizzagalli F. Scartozzi A. Chern A. Kotz S. A. (2022). Identifying a brain network for musical rhythm: a functional neuroimaging meta-analysis and systematic review. *Neurosci. Biobehav. Rev.* 136:104588. 10.1016/j.neubiorev.2022.104588 35259422 PMC9195154

[B44] Keller P. E. Repp B. H. (2008). Multilevel coordination stability: integrated goal representations in simultaneous intra-personal and inter-agent coordination. *Acta Psychol.* 128 378–386. 10.1016/j.actpsy.2008.03.012 18486931 PMC2570261

[B45] Koenig T. Prichep L. Dierks T. Hubl D. Wahlund L. O. John E. R. (2005). Decreased EEG synchronization in Alzheimer’s disease and mild cognitive impairment. *Neurobiol. Aging* 26 165–171. 10.1016/j.neurobiolaging.2004.03.008 15582746

[B46] Kotz S. A. Brown R. M. Schwartze M. (2016). Cortico-striatal circuits and the timing of action and perception. *Curr. Opin. Behav. Sci.* 8 42–45. 10.1016/j.cobeha.2016.01.010

[B47] Kuhl P. K. Ramirez R. R. Bosseler A. Lin J.-F. L. Imada T. (2014). Infants’ brain responses to speech suggest Analysis by Synthesis. *Proc. Natl. Acad. Sci.* 111 11238–11245. 10.1073/pnas.1410963111 25024207 PMC4128155

[B48] Large E. W. Jones M. R. (1999). The dynamics of attending: how people track time-varying events. *Psychol. Rev.* 106 119–159. 10.1037/0033-295X.106.1.119

[B49] Lombard E. (1911). Le signe de l’élévation de la voix. *Annales Des Maladies de L’Oreille et Du Larynx* 37 101–119.

[B50] London J. (2004). *Hearing in Time.* New York, NY: Oxford University Press.

[B51] Madison G. (2006). Experiencing Groove Induced by Music: consistency and Phenomenology. *Music Percept.* 24 201–208. 10.1525/mp.2006.24.2.201

[B52] Madison G. Gouyon F. Ullén F. Hörnström K. (2011). Modeling the tendency for music to induce movement in humans: first correlations with low-level audio descriptors across music genres. *J. Exp. Psychol. Hum. Percept. Perform.* 37 1578–1594. 10.1037/a0024323 21728462

[B53] Manning F. Schutz M. (2013). “Moving to the beat” improves timing perception. *Psychon. Bull. Rev.* 20 1133–1139. 10.3758/s13423-013-0439-7 23670284

[B54] McAuley J. D. Jones M. R. Holub S. Johnston H. M. Miller N. S. (2006). The time of our lives: life span development of timing and event tracking. *J. Exp. Psychol.: Gen.* 135 348–367. 10.1037/0096-3445.135.3.348 16846269

[B55] Merchant H. Honing H. (2014). Are non-human primates capable of rhythmic entrainment? Evidence for the gradual audiomotor evolution hypothesis. *Front. Neurosci.* 7:274. 10.3389/fnins.2013.00274 24478618 PMC3894452

[B56] Miall R. C. (2003). Connecting mirror neurons and forward models. *NeuroReport* 14 2135–2137. 10.1097/00001756-200312020-00001 14625435

[B57] Miyake I. (1902). Researches on rhythmic activity. Studies From the Yale Psychological Laboratory. 10th ed. New Haven, CO: Yale University, 1–48.

[B58] Morillon B. Baillet S. (2017). Motor origin of temporal predictions in auditory attention. *Proc. Natl. Acad. Sc.i U.S.A* 114 E8913–E8921. 10.1073/pnas.1705373114 28973923 PMC5651745

[B59] National Library of Medicine. (2021a). High frequency light and sound stimulation to improve brain functions in Alzheimer’s disease. Available online at: https://clinicaltrials.gov/ct2/show/NCT04042922

[B60] National Library of Medicine. (2021b). Daily light and sound stimulation to improve brain functions in Alzheimer’s disease. Available online at: https://clinicaltrials.gov/ct2/show/NCT04055376

[B61] Niell C. M. Stryker M. P. (2010). Modulation of Visual Responses by Behavioral State in Mouse Visual Cortex. *Neuron* 65 472–479. 10.1016/j.neuron.2010.01.033 20188652 PMC3184003

[B62] Nombela C. Hughes L. E. Owen A. M. Grahn J. A. (2013). Into the groove: can rhythm influence Parkinson’s disease? *Neurosc. Biobehav. Rev.* 37 2564–2570. 10.1016/j.neubiorev.2013.08.003 24012774

[B63] Nozaradan S. Peretz I. Missal M. Mouraux A. (2011). Tagging the Neuronal Entrainment to Beat and Meter. *J. Neurosci.* 31 10234–10240. 10.1523/JNEUROSCI.0411-11.2011 21753000 PMC6623069

[B64] Nozaradan S. Peretz I. Mouraux A. (2012). Selective Neuronal Entrainment to the Beat and Meter Embedded in a Musical Rhythm. *J. Neurosci.* 32 17572–17581. 10.1523/JNEUROSCI.3203-12.2012 23223281 PMC6621650

[B65] Nozaradan S. Schönwiesner M. Caron-Desrochers L. Lehmann A. (2016). Enhanced brainstem and cortical encoding of sound during synchronized movement. *NeuroImage* 142 231–240. 10.1016/j.neuroimage.2016.07.015 27397623

[B66] Pabst A. Balasubramaniam R. (2018). Trajectory formation during sensorimotor synchronization and syncopation to auditory and visual metronomes. *Exp. Brain Res.* 236 2847–2856. 10.1007/s00221-018-5343-y 30051262

[B67] Patel A. D. (2018). *Music as a Transformative Technology of the Mind: An Update. The Origins of Musicality.* Cambridge: MIT Press

[B68] Patel A. D. (2021). Vocal learning as a preadaptation for the evolution of human beat perception and synchronization. *Phil. Trans. R. Soc. B.* 376:20200326. 10.1098/rstb.2020.0326 34420384 PMC8380969

[B69] Patel A. D. Iversen J. R. (2014). The evolutionary neuroscience of musical beat perception: the Action Simulation for Auditory Prediction (ASAP) hypothesis. *Front. Syst. Neurosci.* 8:57. 10.3389/fnsys.2014.00057 24860439 PMC4026735

[B70] Patel A. D. Iversen J. R. Bregman M. R. Schulz I. (2009). Studying Synchronization to a Musical Beat in Nonhuman Animals. *Ann. N. Y. Acad. Sci.* 1169 459–469. 10.1111/j.1749-6632.2009.04581.x 19673824

[B71] Pfordresher P. Q. (2011). “Poor pitch singing as an inverse model deficit: Imitation and estimation,” in *Proceedings of the International Symposium on Performance Science* (Utrecht, the Netherlands: Association Européenne des Conservatoires, Jyväskylä, Finland), 539–544.

[B72] Pfordresher P. Q. Mantell J. (2009). “Singing as a form of vocal imitation: Mechanisms and deficits,” in *Proceedings of the 7th Triennial Conference of European Society for the Cognitive Sciences of Music*, 425–430.

[B73] Phillips-Silver J. Trainor L. J. (2005). Feeling the Beat: movement Influences Infant Rhythm Perception. *Science* 308 1430–1430. 10.1126/science.1110922 15933193

[B74] Phillips-Silver J. Trainor L. J. (2007). Hearing what the body feels: auditory encoding of rhythmic movement. *Cognition* 105 533–546. 10.1016/j.cognition.2006.11.006 17196580

[B75] Pollok B. Rothkegel H. Schnitzler A. Paulus W. Lang N. (2008). The effect of rTMS over left and right dorsolateral premotor cortex on movement timing of either hand. *Eur. J. Neurosci.* 27 757–764. 10.1111/j.1460-9568.2008.06044.x 18279328

[B76] Press C. Cook R. (2015). Beyond action-specific simulation: domain-general motor contributions to perception. *Trends Cogn. Sci.* 19 176–178. 10.1016/j.tics.2015.01.006 25707786

[B77] Prinz W. (1997). Perception and Action Planning. *Eur. J. Cogn. Psychol.* 9 129–154. 10.1080/713752551

[B78] Prinz W. A. (1990). “Common Coding Approach to Perception and Action,” in *Relationships Between Perception and Action*, eds Neumann O. Prinz W. (Berlin, Heidelberg: Springer Berlin Heidelberg), 167–201. 10.1007/978-3-642-75348-0_7

[B79] Rauschecker J. P. (2011). An expanded role for the dorsal auditory pathway in sensorimotor control and integration. *Hear. Res.* 271 16–25. 10.1016/j.heares.2010.09.001 20850511 PMC3021714

[B80] Repp B. H. (2005b). Sensorimotor synchronization: a review of the tapping literature. *Psychonom. Bull. Rev.* 12 969–992. 10.3758/BF03206433 16615317

[B81] Repp B. H. (2005a). Rate Limits of On-Beat and Off-Beat Tapping With Simple Auditory Rhythms. *Music Percept.* 23 165–188. 10.1525/mp.2005.23.2.165

[B82] Repp B. H. Su Y.-H. (2013). Sensorimotor synchronization: a review of recent research (2006–2012). *Psychon. Bull. Rev.* 20 403–452. 10.3758/s13423-012-0371-2 23397235

[B83] Ross J. M. Balasubramaniam R. (2014). Physical and neural entrainment to rhythm: human sensorimotor coordination across tasks and effector systems. *Front. Hum. Neurosci.* 8:576. 10.3389/fnhum.2014.00576 25136306 PMC4118030

[B84] Ross J. M. Comstock D. C. Iversen J. R. Makeig S. Balasubramaniam R. (2022). Cortical mu rhythms during action and passive music listening. *J. Neurophysiol.* 127 213–224. 10.1152/jn.00346.2021 34936516 PMC8794057

[B85] Ross J. M. Iversen J. R. Balasubramaniam R. (2016b). Motor simulation theories of musical beat perception. *Neurocase* 22 558–565. 10.1080/13554794.2016.1242756 27726485

[B86] Ross J. M. Iversen J. R. Balasubramaniam R. (2018). The Role of Posterior Parietal Cortex in Beat-based Timing Perception: a Continuous Theta Burst Stimulation Study. *J. Cogn. Neurosci.* 30 634–643. 10.1162/jocn_a_01237 29346017

[B87] Ross J. M. Warlaumont A. S. Abney D. H. Rigoli L. M. Balasubramaniam R. (2016a). Influence of musical groove on postural sway. *J. Exp. Psychol.: Hum. Percept. Perform.* 42 308–319. 10.1037/xhp0000198 26727019

[B88] Saleh M. Reimer J. Penn R. Ojakangas C. L. Hatsopoulos N. G. (2010). Fast and slow oscillations in human primary motor cortex predict oncoming behaviorally relevant cues. *Neuron* 65 461–471. 10.1016/j.neuron.2010.02.001 20188651 PMC3199221

[B89] Saltuklaroglu T. Bowers A. Harkrider A. W. Casenhiser D. Reilly K. J. Jenson D. E. (2018). EEG mu rhythms: rich sources of sensorimotor information in speech processing. *Brain nd Lang.* 187 41–61. 10.1016/j.bandl.2018.09.005 30509381

[B90] Santarnecchi E. Muller T. Rossi S. Sarkar A. Polizzotto N. R. Rossi A. (2016). Individual differences and specificity of prefrontal gamma frequency-tACS on fluid intelligence capabilities. *Cortex* 75 33–43. 10.1016/j.cortex.2015.11.003 26707084

[B91] Schubotz R. I. (2007). Prediction of external events with our motor system: towards a new framework. *Trends n Cogn. Sci.* 11 211–218. 10.1016/j.tics.2007.02.006 17383218

[B92] Schubotz R. I. Friederici A. D. von Cramon D. (2000). . Time Perception and Motor Timing: a Common Cortical and Subcortical Basis Revealed by fMRI. . *NeuroImage* 11 1–12. 10.1006/nimg.1999.0514 10686112

[B93] Shin Y. K. Proctor R. W. Capaldi E. J. A. (2010). review of contemporary ideomotor theory. *Psychol. Bull.* 136 943–974. 10.1037/a0020541 20822210

[B94] Snyder J. S. Large E. W. (2005). Gamma-band activity reflects the metric structure of rhythmic tone sequences. *Cogn. Brain Res.* 24 117–126. 10.1016/j.cogbrainres.2004.12.014 15922164

[B95] Sperry R. W. (1952). Neurology and the mind-brain problem. *Am. Sci.* 40 291–312.

[B96] Staddon J. (2005). Interval timing: memory, not a clock. *Trends n Cogn. Sci.* 9 312–314. 10.1016/j.tics.2005.05.013 15953755

[B97] Stepp N. Turvey M. T. (2017). Anticipation in manual tracking with multiple delays. *J. Exp. Psychol. Hum. Percept. Perform.* 43 914–925. 10.1037/xhp0000393 28230398

[B98] Stupacher J. Hove M. J. Novembre G. Schütz-Bosbach S. Keller P. E. (2013). Musical groove modulates motor cortex excitability: a TMS investigation. *Brain nd Cogn.* 82 127–136. 10.1016/j.bandc.2013.03.003 23660433

[B99] Su Y.-H. Pöppel E. (2012). Body movement enhances the extraction of temporal structures in auditory sequences. *Psychol. Res.* 76 373–382. 10.1007/s00426-011-0346-3 21695472

[B100] Teki S. Grube M. Griffiths T. D. (2012). A Unified Model of Time Perception Accounts for Duration-Based and Beat-Based Timing Mechanisms. *Front. Integr. Neurosci.* 5:90. 10.3389/fnint.2011.00090 22319477 PMC3249611

[B101] Teki S. Grube M. Kumar S. Griffiths T. D. (2011). Distinct Neural Substrates of Duration-Based and Beat-Based Auditory Timing. *J. Neurosci.* 31 3805–3812. 10.1523/JNEUROSCI.5561-10.2011 21389235 PMC3074096

[B102] Thaut M. H. McIntosh G. C. Rice R. R. Miller R. A. Rathbun J. Brault J. M. (1996). Rhythmic auditory stimulation in gait training for Parkinson’s disease patients. *Mov. Disord.* 11 193–200. 10.1002/mds.870110213 8684391

[B103] Therrien A. S. Lyons J. Balasubramaniam R. (2012). Sensory Attenuation of Self-Produced Feedback: the Lombard Effect Revisited. *PLoS One* 7:e49370. 10.1371/journal.pone.0049370 23145166 PMC3493519

[B104] Tian X. Poeppel D. (2010). Mental imagery of speech and movement implicates the dynamics of internal forward models. *Front. Psychol.* 1:166. 10.3389/fpsyg.2010.00166 21897822 PMC3158430

[B105] Torre K. Balasubramaniam R. (2009). Two different processes for sensorimotor synchronization in continuous and discontinuous rhythmic movements. *Exp. Brain Res.* 199 157–166. 10.1007/s00221-009-1991-2 19711062

[B106] van Noorden L. Moelants D. (1999). Resonance in the Perception of Musical Pulse. *J. N. Music Res.* 28 43–66. 10.1076/jnmr.28.1.43.3122

[B107] Varlet M. Nozaradan S. Trainor L. Keller P. E. (2020). Dynamic Modulation of Beta Band Cortico-Muscular Coupling Induced by Audio-Visual Rhythms. *Cereb. Cortex Commun.* 1:tgaa043. 10.1093/texcom/tgaa043 34296112 PMC8263089

[B108] Ventura M. I. Barnes D. E. Ross J. M. Lanni K. E. Sigvardt K. A. Disbrow E. A. A. (2016). pilot study to evaluate multi-dimensional effects of dance for people with Parkinson’s disease. *Contemp. Clin. Trials* 51 50–55. 10.1016/j.cct.2016.10.001 27765693 PMC5108673

[B109] Vorberg D. Wing A. (1996). Chapter 4 Modeling variability and dependence in timing. *Handbook of Perception and Action* 2 181–262. 10.1016/S1874-5822(06)80007-1

[B110] Vos P. G. Helsper E. L. (1992). “Tracking Simple Rhythms: On-Beat Versus Off-Beat Performance,” in *Time, Action and Cognition*, eds Macar F. Pouthas V. Friedman W. J. (Dordrecht: Springer Netherlands), 287–299. 10.1007/978-94-017-3536-0_30

[B111] Vuust P. Dietz M. J. Witek M. Kringelbach M. L. (2018). Now you hear it: a predictive coding model for understanding rhythmic incongruity: now you hear it. *Ann. N. Y. Acad. Sci.* 1423 19–29. 10.1111/nyas.13622 29683495

[B112] Vuust P. Ostergaard L. Pallesen K. J. Bailey C. Roepstorff A. (2009). Predictive coding of music – Brain responses to rhythmic incongruity. *Cortex* 45 80–92. 10.1016/j.cortex.2008.05.014 19054506

[B113] Wekselblatt J. B. Niell C. M. (2015). Behavioral State—Getting “In The Zone.”. *Neuron* 87 7–9. 10.1016/j.neuron.2015.06.020 26139365 PMC4907540

[B114] Wolpert D. M. Flanagan J. R. (2016). Computations underlying sensorimotor learning. *Curr. Opin. Neurobiol.* 37 7–11. 10.1016/j.conb.2015.12.003 26719992 PMC6103431

[B115] Wolpert D. M. Flanagan J. R. Wolpert D. M. Flanagan J. R. (2009). “Forward models,” in *The Oxford Companion to Consciousness*, eds Baynes T. Cleeremans A. Wilken P. (Oxford: Oxford University Press).

[B116] Wolpert D. M. Kawato M. (1998). Multiple paired forward and inverse models for motor control. *Neural Netw.* 11 1317–1329. 10.1016/S0893-6080(98)00066-5 12662752

[B117] Woodrow H. (1932). The effect of rate of sequence upon the accuracy of synchronization. *J. Exp. Psychol.* 15 357–379. 10.1037/h0071256

[B118] Yang S. C.-H. Wolpert D. M. Lengyel M. (2016). Theoretical perspectives on active sensing. *Curr. Opin. Behav. Sci.* 11 100–108. 10.1016/j.cobeha.2016.06.009 30175197 PMC6116896

[B119] Zatorre R. J. Chen J. L. Penhune V. B. (2007). When the brain plays music: auditory–motor interactions in music perception and production. *Nat. Rev. Neurosci.* 8 547–558. 10.1038/nrn2152 17585307

[B120] Zollinger S. A. Brumm H. (2011). The Lombard effect. *Curr. Biol.* 21 R614–R615. 10.1016/j.cub.2011.06.003 21854996

